# Multi-Country Evaluation of Safety of Dihydroartemisinin/Piperaquine Post-Licensure in African Public Hospitals with Electrocardiograms

**DOI:** 10.1371/journal.pone.0164851

**Published:** 2016-10-20

**Authors:** Abdunoor M. Kabanywanyi, Rita Baiden, Ali M. Ali, Muhidin K. Mahende, Bernhards R. Ogutu, Abraham Oduro, Halidou Tinto, Margaret Gyapong, Ali Sie, Esperanca Sevene, Eusebio Macete, Seth Owusu-Agyei, Alex Adjei, Guillaume Compaoré, Innocent Valea, Isaac Osei, Abena Yawson, Martin Adjuik, Raymond Akparibo, Mwaka A. Kakolwa, Salim Abdulla, Fred Binka

**Affiliations:** 1 Ifakara Health Institute, Dar es Salaam, Tanzania; 2 INDEPTH Network, Accra, Ghana; 3 Navrongo Health Research Centre, Navrongo, Ghana; 4 Nanoro Health Research Centre, Nanoro, Burkina Faso; 5 Dodowa Health Research Centre, Dodowa, Ghana; 6 Nouna Health Research Centre, Nouna, Burkina Faso; 7 Centro de Investigaçãoem Saúde de Manhiça(CISM), Manhiça, Mozambique; 8 Kintampo Health Research Centre, Kintampo, Ghana; 9 University for Health and Allied Sciences, Ho, Ghana; Centers for Disease Control and Prevention, UNITED STATES

## Abstract

The antimalarial drug piperaquine is associated with delayed ventricular depolarization, causing prolonged QT interval (time taken for ventricular de-polarisation and re-polarisation). There is a lack of safety data regarding dihydroartemisinin/piperaquine (DHA/PPQ) for the treatment of uncomplicated malaria, which has limited its use. We created a platform where electrocardiograms (ECG) were performed in public hospitals for the safety assessment of DHA/PPQ, at baseline before the use of dihydroartemisinin/piperaquine (Eurartesim^®^), and on day 3 (before and after administration of the final dose) and day 7 post-administration. Laboratory analyses included haematology and clinical chemistry. The main objective of the ECG assessment in this study was to evaluate the effect of administration of DHA/PPQ on QTc intervals and the association of QTc intervals with changes in blood biochemistry, full and differential blood count over time after the DHA/PPQ administration. A total of 1315 patients gave consent and were enrolled of which 1147 (87%) had complete information for analyses. Of the enrolled patients 488 (42%), 323 (28%), 213 (19%) and 123 (11%) were from Ghana, Burkina Faso, Tanzania and Mozambique, respectively. Median (lower—upper quartile) age was 8 (5–14) years and a quarter of the patients were children under five years of age (n = 287). Changes in blood biochemistry, full and differential blood count were temporal which remained within clinical thresholds and did not require any intervention. The mean QTcF values were significantly higher than on day 1 when measured on day 3 before and after administration of the treatment as well as on day 7, four days after completion of treatment (12, 22 and 4 higher, p < 0.001). In all age groups the values of QT, QTcF and QTcB were highest on day 3 after drug intake. The mean extreme QTcF prolongation from baseline was lowest on day 3 before drug intake (33 ms, SD = 19) and highest on day 3 after the last dose (60 ms, SD = 31). There were 79 (7%) events of extreme mean QTcF prolongation which were not clinically significant. Nearly a half of them (n = 37) were grade 3 and mainly among males (33/37). Patients in Burkina Faso, Mozambique and Tanzania had significantly lower mean QTcF than patients in Ghana by an average of 3, 4 and 11 ms, respectively. We found no evidence that Eurartesim^®^ administered in therapeutic doses in patients with uncomplicated malaria and no predisposing cardiac conditions in Africa was associated with adverse clinically significant QTc prolongation.

## Introduction

Dihydroartemisinin/piperaquine (DHA/PPQ) is one of the artemisinin based combination therapies (ACTs) effective against uncomplicated malaria. Its use has been limited due reported cardiotoxic effects [1]. Currently there is limited data on cardiac effects to warrant wider deployment of DHA/PPQ. One of the side effects of aminoquinolone group in which piperaquine, a part of DHA/PPQ combination belongs is the negative effect on heart rhythm through prolongation of QT intervals [2]. The QT interval is the time from the start of the Q wave to the end of the T wave and represents the time taken for ventricular de-polarisation and re-polarisation.

The QT prolongation effect can be exacerbated in individuals that are predisposed to a form of fatal tachy-arrhythmias called *torsades de pointes* (TdP). In clinical pharmacology prolonged QT interval is a heart rhythm’s toxicity indicator recorded upon confirmation of Fridericia’s correction > 450 milliseconds [3,4]. In clinical literature according to the Committee for Proprietary Medical Products (CPMP) criteria, female adults and male adults are considered to have a prolonged QT intervals if the calculated Bazzet-correction exceeds 470 and 450ms, respectively [5]. It is further suggested by CPMP that while 450ms in adult females and 430ms in adult males are normal values, values close to 451–470ms in adult females and 431–450ms in adults males are considered borderline [5]. In 2012 the International Conference on Harmonisation of Technical Requirements for Registration of Pharmaceuticals for Human Use (ICH) in collaboration with Food and Drug Administration of United states of America (FDA) issued revised guidance for Industry (E14) on clinical evaluation of electrocardiographic “Q” and “T” waves’ (QT/calculated-QTc) interval prolongation for Non-Antiarrhythmic drugs [5]. In this guidance, details of using Bazett or Fridericia (B/F) formulae for calculated (QTcB/F) is also presented. It was recommended that outliers be categorized as QTc prolongation adverse events: Grade 1 > 450ms, Grade 2 > 480ms, and Grade 3 > 500ms, respectively regardless of gender as opposed to the normal values that would necessarily be investigated in healthy volunteers [6]. This paper applied similar cut-off points and defines QTc increase of 30ms and above from baseline as QTc with extreme prolongation.

In DHA/PPQ the water soluble dihydroartemisinin (DHA) is rapidly absorbed and responsible for enhanced efficacy of the ACT, suggesting an additive antimalarial effect [7]. Piperaquine (PPQ) the fat-soluble component of this ACT is solely responsible for the delayed ventricular depolarization hence QT prolongation. PPQ is one of the 4-aminoquinoline synthetic alkaloids that kill malaria parasites through intraparasitic detoxification of haem. PPQ is slowly absorbed after single and multiple dosing with maximum time (Tmax) of about 4–5 hours [8]. The slow elimination of PPQ in plasma is associated with its long acting properties and this is the main cause of the QT interval delays particularly in patients prone to QT interval prolongation[1],[9]. In malaria endemic settings there have been no records of fatal QT toxicity incidence following treatment with PPQ [10]. This is most probably due to lack of toxicity especially when this antimalarial is given as optimal therapeutic dose [11]. However, it could also be due to the limited knowledge of side effects that go undetected or reported as the result of a lack of ECG equipment which are expensive to implement and require trained staff to use. Most remote health facilities where a large proportion of uncomplicated malaria is managed do not have ECG machines. Among the few cases of QTc prolongation following administration of PPQ that have been reported to date, most are from clinical efficacy follow up trials in which the full ECG evaluation is limited and there are few subjects in the low age group of children less than 5 years [12,13]. In a recently completed meta-analysis of ACT including DHA/PPQ the emphasis was on efficacy and for DHA/PPQ did not report any safety issues requiring further detailed investigation [14]. In a detailed randomized, double-blind, placebo-controlled clinical trial of DHA/PPQ in Cambodia a regimen of compressed 2-day treatment courses was associated with the prolongation of QTcF >500ms among male military servicemen prompting a halt after nearly two months of implementation and suggesting further more thorough evaluation with patients in malaria endemic areas [15]. This trial reported that increased repolarization risk was associated with repeated and compressed 2 dose regimen, and that this might be mitigated by using a conventional 3-day regimen, with patients given medication on empty stomach and avoiding repeated dosing or co-administration with other QT-prolonging medications [15].

Here we report an investigation of safety of DHA/PPQ with ECG in patients of all age groups who presented in public health facilities with un-complicated *Plasmodium faciparum* malaria in sub-Saharan Africa.

## Materials and Methods

### Participants’ enrolment

We conducted a post-licensure clinical study to assess safety of DHA/PPQ (Eurartesim^®^) in health facilities within the International Network for the Demographic Evaluation of Populations and Their Health (INDEPTH) [16] over a period of 10 months between October 2013 and July 2014, S1 Checklist and S1 Protocol. The main objective of the ECG assessment in this study was to evaluate the relationship of the effect resulted from the administration of DHA/PPQ on QTc intervals and the association of QTc intervals with changes in blood biochemistry, full and differential blood count over time after the DHA/PPQ administration.

1315 Patients were enrolled at out-patient Departments (OPDs) from Manhiça Mozambique, Rufiji Tanzania, in three Ghanaian sites; Navrongo, Kintampo and Dodowa as well as in Nouna and Nanoro both in Burkina Faso. All eligible patients provided written informed consent and assent as appropriate, we also obtained consent from parents or guardians of the minors (<18 years) that were included in the study.

The inclusion criteria were: 1) willingness to participate in the study, 2) age above 6 months and 3) provision of consent. Exclusion criteria were: 1) severe malaria, 2) known allergy to artemisinin or piperaquine, 3) known pregnancy, 4) lactating women and 5) taking medicinal products that are known to prolong the QTc interval (Table 1), 6) having taken a DHA/PPQ dose in the previous four weeks, 7) family history of sudden unexplained death, or 8) personal or family history of predisposing cardiac conditions for arrhythmia/QT prolongation (including congenital long QT syndrome, arrhythmia, and any known QTc interval greater than 450 milliseconds with either Bazett or Fridericia correction).

**Table 1:** Products that are known to prolong the QTc interval.

Group	Type of medicinal product
**Antiarrhythmics**	Amiodarone, disopyramide, dofetilide, ibutilide, procainamide, quinidine, hydroquinidine, sotalol
**Neuroleptics**	Phenothiazines, sertindole, sultopride, chlorpromazine, haloperidol, mesoridazine, pimozide, or thioridazine), antidepressive agents
**Certain antimicrobial agents**	Macrolides (e.g. erythromycin, clarithromycin) Fluoroquinolones (e.g. moxifloxacin, sparfloxacin), Imidazole and triazole antifungal agents pentamidine and saquinavir
**Certain non-sedating antihistamines**	Terfenadine, astemizole, mizolastine.
**Antimalarials**	Mefloquine, halofantrine, lumefantrine, chloroquine, quinine and other aminoquinoline agents
**Others**	Cisapride, droperidol, domperidone, bepridil, diphemanil, probucol, levomethadyl, methadone, vinca alkaloids, arsenic trioxide

### ECG procedure

ECGs were recorded using the 12-lead data collection recorder (Mortara ELI 150) supplied in collaboration with *CardiaBase SAS* headquartered in Nancy, France [17]. *CardiaBase* is a leading company in the reading and interpretation of cardiac data with a particular focus on early phases of clinical trials complying with FDA requirements. ECG ELI files were uploaded directly to specific computer directory called ELI and sent to *CardiaBase*. All analytical feedback from *CardiaBase* was sent back to study sites in PDFs. In addition, for each file detailed QTcF counts were provided to clinicians for compilation and appropriate medical action required by the participants during DHA/PPQ administration and follow up period.

During ECG, patients were in a relaxed supine position for at least 5–10 minutes before recording. Patients were further encouraged to breathe normally and remain awake during the procedure. They were also instructed to remain silent and not touch anything especially metallic objects during the recording. All laboratory procedures (blood draws and malaria Rapid Diagnostic [mRDTs] testing) and blood pressure measurements were performed after the ECG recording was completed. Younger children remained with their parents in the examination rooms. Clinicians unplugged the machine from AC power which was battery operated during recording to avoid contact with metallic objects. The 12 chest leads positioning were standardised across sites for all ECG recording. Electrode cables were joined on to a single use electrode cell using the International Electrotechnical Commission-IEC or Association for the Advancement of Medical Instrumentation-AAMI placement systems. Electrodes were placed on the chest as follows; (C1 or V1) on the fourth intercostal space at the right sternal border; (C2 or V2) on the fourth intercostal space at the left sternal border; (C3 or V3) on the midway between V2 and V4; (C4 or V4) on the fifth intercostal space at the left of the mid-clavicular line; (C5 or V5) along the anterior axillary line at same horizontal level as V4 and (C6 or V6) along the mid-axillary line on same horizontal level as V4 and V5. The limb connections were; (R or RA) on the right deltoid fossa, mid-clavicular; (L or LA) on the left deltoid fossa midclavicular; for both upper limbs whereas (N or RL) on the right anterior axillary, mid-clavicular line and (F or LL) on the left anterior axillary, mid-clavicular line connected lower limbs.

ECG examinations were recorded on Day 1 (before drug administration), twice on Day 3 (i.e. before and 3–4 hours after the last dose administration) as well as on Day 7. The QTcF (using Fridericia’s cube-root formula) intervals at baseline were considered normal upon confirmation of the calculated average QTcF recording of ≤450ms in males and ≤470ms in females. All ECGs were collected in singly with the exception of ECGs taken on Day 1 and Day 3 after the last dose administration, which were recorded in triplicate. In case of any abnormality in the QTcF value assessed on baseline before drug intake, DHA/PPQ was withheld until QTcF returned to normal values, thereafter the DHA/PPQ dose was to be completed under frequent QTc monitoring based on investigator clinical judgment. If the QTcF did not return below 480 ms in male or below 500 ms in female within 6 hours of the DHA/PPQ administration, another antimalarial therapy was initiated and patient was observed for till the QTcF returned to normal value during the 28 day follow up.

### Drug administration

Eurartesim^®^ was administered every 24 hours from the first administration over a period of three days, i.e. on Day 1, then after 24 hours (Day 2) and after 48 hours (Day 3). The dosage was based on patient’s body weight. Two strengths of Eurartesim^®^ dosing strengths were used; 20/160mg and 40/320mg of DHA and PQP for children and adults respectively. The drug was administered with water on empty stomach (no food at least three hours before or three hours after DHA/PPQ intake). To facilitate drug administration in small children, tablets were crushed on a spoon and given with water. For those who vomited within 30 min of drug administration, the dose was re-administered. For vomiting which occurred between 30 and 60 min after administration a half dose was re-administered. Re-dosing was not attempted more than once. In case of vomiting of rescue repeat dose a rescue treatment was offered immediately. Research team members assessed completion of the full treatment course during home visits and counted the number of pills remaining to check patient compliance.

### Laboratory tests

*Plasmodium falciparum* microscopy with thick and thin blood smears was performed for screening of eligible participants. Thick smear was used for parasite counts and the thin smears for identification of *Plasmodium* species. Parasite count per 200 white blood cells was used to calculate parasite density. Any subject found with mixed infections was withdrawn. Venous phlebotomy was conducted to collect blood for haematology and biochemistry analyses during scheduled patients visits on Day 1, day 3 (before and after drug administration) as well as on day 7. Plasma samples were prepared to evaluate: Potassium, Chloride, Urea, ALAT, ASAT and total Bilirubin. Hematology was performed to evaluate haemoglobin and cell count. Full and differential blood counts were routinely analyzed using semi-automated and fully-automated Sysmex haematology machines (Sysmex Europe GmbH) [18]. For blood biochemistry we used Cobas machine (Roche Diagnostics, Roche Group, Basel Switzerland) [19].

### Sample size determination

The sample size computation was based on prolonged QTc interval as the major manifestation of adverse events related to cardio-toxicity following intake of DHA/PPQ. It was therefore hypothesized that there will be a probability of 0.95 to observe at least one cardiac adverse event of QT prolongation assuming that the true incidence of these events is 3 per 1000 patients treated with precision of 0.002 and 95% confidence interval. For such probability therefore nearly 1,000 subjects were to be enrolled for close follow up with ECG measurements.

### Statistical analysis

Summary statistics were calculated to describe the data set and variable distribution. Categorical data were reported using numbers and percentages whereas continuous data was summarized through median and interquartile range (IQR) or mean and standard deviation (SD) depending on the data distribution. While continuous background demographic information was presented in median and IQR by age stratification, the mean biochemistry values across age groups were presented with SD. Stratification by age groups was also applied in summary statistics for electrocardiographic readings where each follow up visit was considered as independent. QTcF/B was measured on pre-treatment during Day 1, during treatment on day 3 (pre and post treatment) and on day 7 after treatment course.

QTcF/B on day 3 (before and after treatment) was measured in triplicate and the average of the three measurements was taken to represent the measurement on that time (day 3 before and after treatment). The Skillings–Mack statistic (Friedman test accounting for missing value) was used to compare the median of more than two groups.

Events of mean extreme QTc prolongation was based on those patients who had the mean QTcF above 450ms (male) or 470ms (female) at any point of measurement from baseline following administration of Eurartesim^®^. The mean prolongation was then calculated by taking the average difference of QTc in the follow up visits and QTc at baseline. The number of events was calculated as absolute number of patients who had extreme QTc prolongation above 450 (male) or 470 (female). Grade 1 cardiac event of QTc prolongation was defined as QTc increase from baseline (above 450 for male and 470 for female) between 0–30ms. Grade 2 was defined as the increase in QTc from 31–50ms and Grade 3 was the increase in QTc above 50ms from baseline.

A linear mixed model was fitted to determine the relationship between QTcF and risk factors accounting for between subject variability. A likelihood ratio (LR) test was used to compare the linear mixed model with and without random slope and was also used to investigate the effect of each covariate to be included into the final model. As the QTcF interval was expected to differ between children and adults, a model with different slopes for each age groups was fitted and compared with models having similar slope. This was done using both the likelihood ratio test and Bayesian Information Criteria (BIC). The effect of age (categorical variable) and day on the QTcF was kept in the model from the beginning of model development. Independent variables tested were age, gender, haemoglobin, total bilirubin, ALT, AST, creatinine, BUN, potassium, chloride, day of blood sampling, body mass index and country. The final model was built on the full data set which was then implemented to account for a complete case data set (the data set with all measurements extracted from all subjects), the same was used in a stratified analysis by country. A user written program gllamm with 15 quadrature points was used to model both random intercept and random slope. This analysis utilized STATA version 13 (Stata Corp, Texas, USA) software. P-value of 0.05 and less was considered statistically significant.

### Ethics approval and registration

Written informed consent for adults and assent for minors was obtained from all patients before study-related activity. Informed consent was also obtained from parents or guardians of the minors included in the study. The protocol was approved by the institutional and national ethics committees in Tanzania (Ifakara Health Institute’ institutional review board and the National ethics committee of the Ministry of Health and Social welfare), Mozambique (Manhiça institutional review board: Comité Institucional de Bioética para Saúde do Centro de Investigação em Saúde da Manhiça and the National ethics committee: Comité Nacional de Bioética para a Saúde), Ghana (Ghanaian Health Service Ethical Review Committee) and Burkina Faso (the national ethics committee: Comite d’Ethique pour la Recherche en santé (CERS)). Dates of approvals differed by country although in all countries relevant approvals were obtained in 2013 prior to enrolment of the first participant in to the trial. The study was registered with Clinical trials.gov on May 1, 2013 (NCT02199951) prior to enrolment of the first subject. Test dugs were outsourced from Sigma Tau in Italy through Medicine for Malaria Venture (MMV) [20]. Eurartesim^®^ was registered in participating countries before use: Tanzania—Food & Drug Authority; Ghana—Food and Drugs Board; Mozambique—Ministério da Saúde Departamento Farmacêutico of the Ministry of Health (MISAU) and in Burkina Faso—Direction Generale de la Pharmacie, du Medicament et des Laboratoire (DGPML) of the Ministry of Health.

## Results

### Clinical and demographic background of study participants

**Fig 1:**
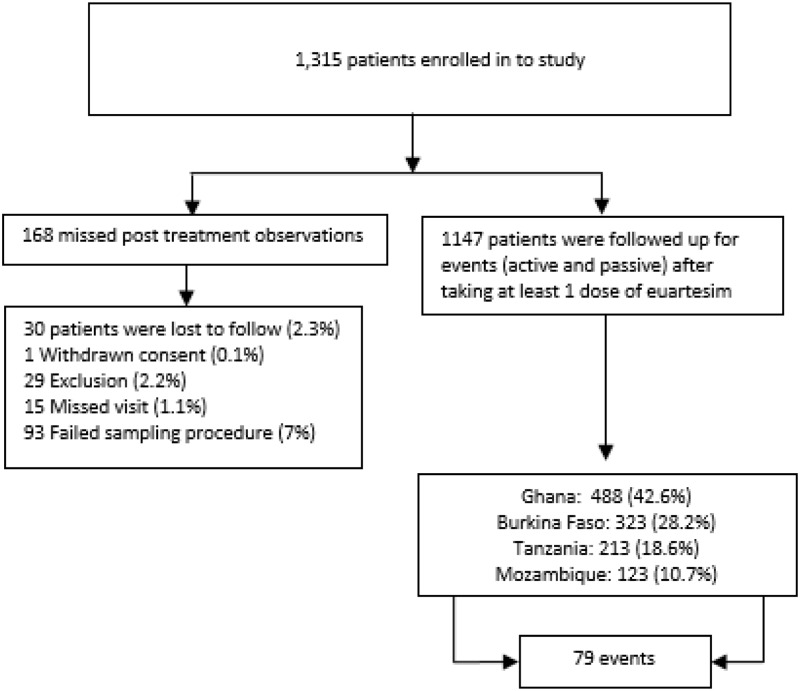
Patients’ flow.

A total of 1315 patients were enrolled in the study. For analysis, we included all subjects who had at least one ECG measurement post treatment (n = 1147; 87%), among whom 1055 subjects had no missing ECG data (the results of the analysis of full data was not different to the analysis of complete data-results not shown). In total, 168 (13%) subjects missed all post treatment observations for different reasons. One subject (0.1%) withdrew consent, 29 (2%) patients were excluded for reasons such as failure of phlebotomy and lack of cooperation, 15 (1%) missed visit, 30 (2%) were Loss To Follow-up (LTFU) and 93 (7%) failed to attend blood sampling and ECG measurements on the prescribed follow up visits or specified time (Fig 1). Out of 1147 patients, 488 (42%) were from Ghana, 323 (28%) were from Burkina Faso, 213 (19%) were from Tanzania and 123 (11%) were from Mozambique. In total, 552 (48%) were male and 595 (52%) were female. Median (lower—upper quartile) age was 8 (5–14) years. A quarter of the patients were children under five years of age (287, 25%). Clinical and demographic background is presented in Table 2. After first dose 31/1147 (3%) vomited within 30 minutes and 7/1147 (0.6%) vomited between 30 minutes and 1 hour. Only 1/1147 (0.1%) patient vomited on the second day and none of them vomited on the third day.

**Table 2:** Clinical and demographic background of study participants. Data presented are Median and Interquartile Range (IQR) for each measurement.

Attributes	Age group*				
**Parameter**	6 months—5 years	5—< 12 years	12—< 18 years	18+ years	Total
	287 (25.0%)	507 (44.2%)	143 (12.5%)	210 (18.3%)	1147
**Age (years)**	3.5 (1.6)	7.6 (3.1)	13.9 (2.0)	30.1 (24.8)	8.1** (8.7)
**Weight (kg)**	12.9 (4)	20.9 (7.3)	40 (15.0)	59.0 (14.0)	21.4 (25.0)
**Height (cm)**	93 (12.0)	121 (19)	151 (18.0)	162.0 (11.5)	124.0 (44.0)
**Haemoglobin (g/dl)**	9.4 (2.1)	10.6 (1.7)	11.6 (1.7)	12.2 (2.1)	10.7 (2.3)

*number of participants and percentage of total cohort by age group

** Median (IQR) for cohort.

### Effect of Eurartesim^®^ on Biochemistry and Haematology

Young children were more anaemic as compared to older children and adults who were least anaemic (Table 2). The median biochemistry values across age groups during Eurartesim^®^ treatment period were recorded low as compared to baseline values on Day 1 (Table 3). Although not completely returned to normal, these values tended to reverse to baseline values by day 7 after completion of the full treatment course. The exception to this was potassium value at baseline was the only value that was recorded higher on day 3 or day 7 than on day 1 (The Skillings–Mack statistic test p-value < 0.05). Creatinine in children was also lower than the acceptable ranges whereas in general all these changes remained within clinical thresholds that did not require further interventions.

**Table 3:** Background and laboratory parameters.

Parameter	Day 1* (n = 1143)	Day 3* (n = 1127)	Day 7* (n = 1121)	Normal values	p-value^a^
**6 months—< 5 years**					
**Bilirubin total (μmoll−1)**	12.1 (12.4)	6.3 (5.2)	7.3 (5.6)	<25.7	< 0.001
**ALT (UL−1)**	28.8 (19.9)	23.7 (14.7)	25.3 (14.7)	Feb-60	< 0.001
**AST (UL−1)**	29.7 (18.4)	26.0 (16.4)	27 (15.9)	15–55	< 0.001
**Creatinine (μmolL−1)**	31.9 (16.7)	29.9 (15.9)	29.0 (13.3)	20–62	< 0.001
**BUN (mmolL−1)**	3.2 (2.3)	2.6 (1.9)	2.5 (1.7)	1.7–8.3	< 0.001
**Potassium (mmolL−1)**	4.1 (0.9)	4.1 (1.1)	4.4 (0.8)	3.5–5.6	< 0.001
**Chloride (mmolL−1)**	102.5 (10.9)	104.0 (9.9)	104.5 (8.2)	96–105	< 0.001
**5—< 12 years**					
**Bilirubin total (μmoll−1)**	12.5 (14.2)	5.7 (5.4)	6.2 (5.0)	<25.7	< 0.001
**ALT (UL−1)**	24.3 (13.5)	22.0 (13.4)	23.0 (13.6)	Feb-60	< 0.001
**AST (UL−1)**	26.5 (160)	24.8 (13.7)	25.0 (13.1)	15–55	< 0.001
**Creatinine (μmolL−1)**	41.0 (21.1)	40.1 (21.7)	39.6 (19.5)	55–97	< 0.001
**BUN (mmolL−1)**	3.9 (3.5)	3.3 (3.1)	3.3 (2.9)	1.7–8.3	< 0.001
**Potassium (mmolL−1)**	4.1 (0.7)	4.1 (0.7)	4.3 (0.6)	3.5–5.6	< 0.001
**Chloride (mmolL−1)**	103 (8.8)	103.7 (8.4)	103 (7.8)	96–105	0.0073
**12—< 18 years**					
**Bilirubin total (μmoll−1)**	15.4 (16.4)	7.3 (5.9)	7.6 (6.3)	<25.7	< 0.001
**ALT (UL−1)**	22.0 (15.2)	22.2(12.6)	23.3 (12.7)	Feb-60	0.7524
**AST (UL−1)**	23.7 (13.4)	23.5 (14.1)	23.5 (12.6)	15–55	0.6303
**Creatinine (μmolL−1)**	55.5 (24.7)	58.7 (28.1)	53.0 (29.1)	55–97	0.0013
**BUN (mmolL−1)**	4.2 (5.6)	3.9 (4.0)	3.1 (3.0)	1.7–8.3	< 0.001
**Potassium (mmolL−1)**	4.1 (0.7)	4.1 (0.7)	4.3 (0.6)	3.5–5.6	< 0.001
**Chloride (mmolL−1)**	101 (9.2)	102.4 (9.5)	102.5 (7.3)	96–105	0.002
**18 + years**					
**Bilirubin total (μmoll−1)**	14.0 (15.3)	8.0 (7.4)	9.1 (7.0)	<25.7	< 0.001
**ALT (UL−1)**	22.9 (17.8)	22.5 (17.2)	23.3 (18.0)	Feb-60	0.1931
**AST (UL−1)**	22.8 (15.1)	24.0 (15.2)	23.7 (13.5)	15–55	0.6815
**Creatinine (μmolL−1)**	71.3 (33.7)	73.2 (33.6)	72.6 (30.2)	55–97	0.1315
**BUN (mmolL−1)**	3.8 (3.9)	4.0 (3.4)	3.8 (3.2)	1.7–8.3	0.3926
**Potassium (mmolL−1)**	4.1 (0.8)	4.1 (0.7)	4.3 (0.8)	3.5–5.6	< 0.001
**Chloride (mmolL−1)**	101.0 (12.5)	103 (9.4)	103 (9.7)	96–105	0.1057

* Values in Median (interquartile range),

^^a^^ The Skillings–Mack statistic p-value comparing median value for each parameter over days.

### Effect of Eurartesim^®^ on QTcF

**Fig 2:**
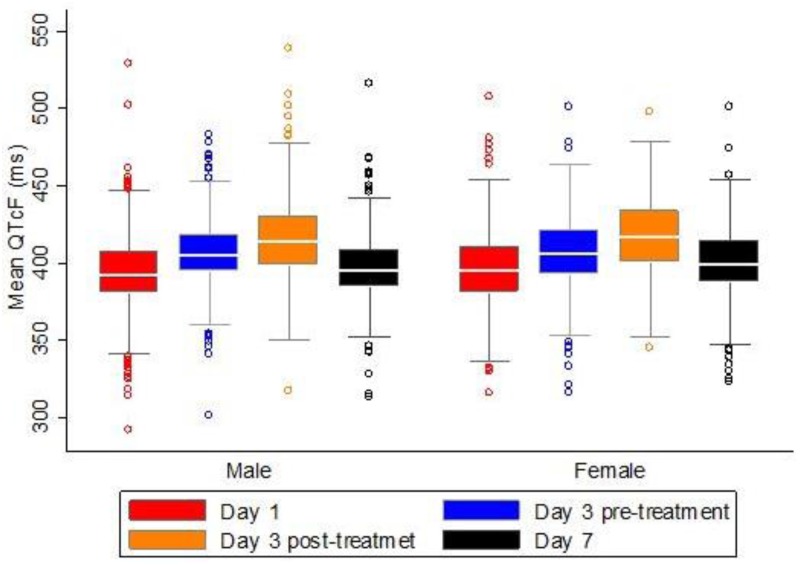
Mean QTcF over time by sex.

**Fig 3:**
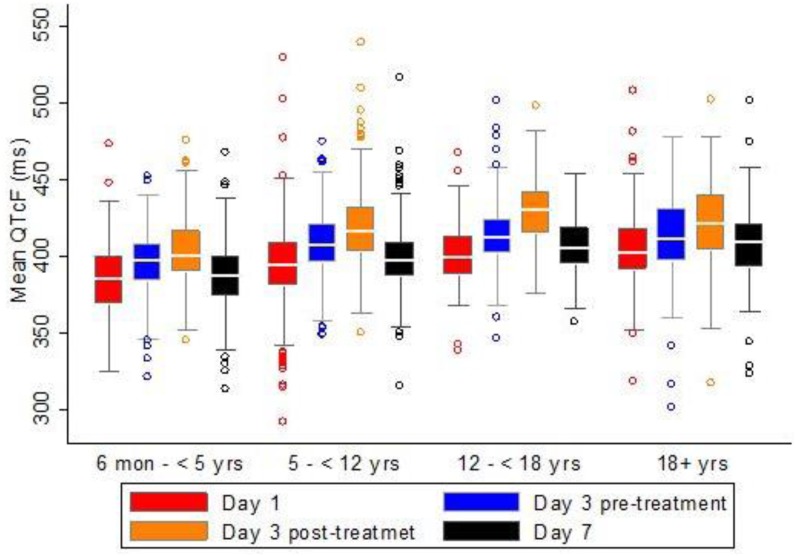
Mean QTcF over time by age groups.

**Fig 4:**
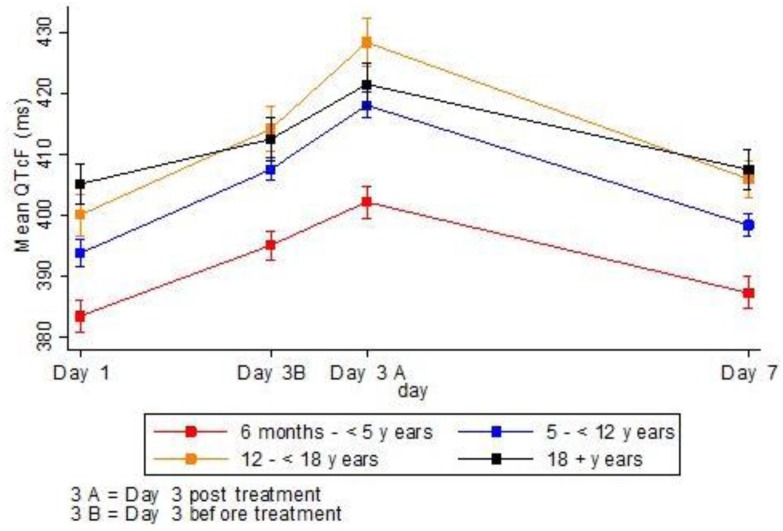
Mean QTcF by age groups over days.

Children less than 5 years of age had a lower mean QTcF (392.2 ± 1.0) as compared with children of older ages; 5–12 years (404.42 ± 0.7), 12–18 years (412.1 ± 1.4) and adults (411.8 ± 1.1), p-value <0.001. At baseline the mean QTcF was the same in male and female subgroups (Fig 2). In all age groups the mean QTcF was increasing during the Eurartesim^®^ treatment period and declined in the later days at the end of treatment course (Figs 3 and 4).

In all age groups the values of QT, QTcF and QTcB were significantly higher on day 3 after drug intake than any other day. R-R was recorded high on day 3 in all age groups after drug intake which was statistically significant different from day 1 (Skillings–Mack statistic test p-value < 0.001). Heart rate following drug intake on day 1 and subsequent days was clinically and statistically differently low as compared to the value on baseline confirming QT prolongation with dosing, p-value *<* 0.001 (Table 4).

**Table 4:** Electrocardiogram readings.

Parameter	Day 1* (n = 1137)	Day 3 before (n = 1132)	Day 3 after (n = 1132)	Day 7 (n = 1069)	p-value^a^
**6 months—5 years**					
**R-R (ms)**	480 (91)	538 (101)	535 (95)	528 (104)	< 0.001
**Heart rate (bpm)**	126 (23)	112 (21)	113 (21)	113 (21)	< 0.001
**QRS(ms)**	72 (6)	72 (7)	73 (7)	72 (6)	< 0.001
**QT (ms)**	301 (39)	322 (32)	325 (38)	315 (33)	< 0.001
**QTcF (ms)**	385 (31)	397 (25)^≠^	400 (27)^≠^	387 (26)	< 0.001
**QTcB (ms)**	434 (25)	440 (23)^≠^	445 (25)^≠^	430 (26)	< 0.001
**5—< 12 years**					
**R-R (ms)**	571 (143)	660 (148)	668 (135)	646 (131)	< 0.001
**Heart rate (bpm)**	105 (26)	91 (20)	90 (19)	92 (19)	< 0.001
**QRS(ms)**	77 (8)	78 (8)	78 (8)	77 (7)	< 0.001
**QT (ms)**	333 (46)	357 (39)^≠^	365 (46)^≠^	346 (35)	< 0.001
**QTcF (ms)**	394 (29)	407 (26)^≠^	417 (29)^≠^	398 (23)	< 0.001
**QTcB (ms)**	431 (26)	437 (28)^≠^	445 (29)^≠^	429 (24)	< 0.001
**12—< 18 years**					
**R-R (ms)**	648 (175)	758 (182)	764 (158)	743 (155)	< 0.001
**Heart rate (bpm)**	93 (25)	79 (19)	78 (16)	80 (17)	< 0.001
**QRS(ms)**	84 (11)	85 (10)	85 (10)	83 (11)	< 0.001
**QT (ms)**	348 (48)	377 (38)^≠^	393 (43)^≠^	369 (33)	< 0.001
**QTcF (ms)**	399 (26)	412 (23)^≠^	430 (28)^≠^	405 (25)	< 0.001
**QTcB (ms)**	428 (20)	433 (29)^≠^	449 (31)^≠^	426 (30)	< 0.001
**18+ years**					
**R-R (ms)**	729 (213)	801 (219)	836 (176)	811 (202)	< 0.001
**Heart rate (bpm)**	82 (22)	75 (19)	71 (16)	73 (17)	< 0.001
**QRS(ms)**	87 (11)	88 (12)	89 (10)	88 (10)	0.0056
**QT (ms)**	365 (47)	390 (44)^≠^	400 (43)^≠^	380 (38)	< 0.001
**QTcF (ms)**	403 (27)	412 (35)^≠^	422 (36)^≠^	409 (28)	< 0.001
**QTcB (ms)**	426 (26)	427 (29)^≠^	435 (33)^≠^	425 (30)	< 0.001

* Values in Median (interquartile range),

^^a^^ The Skillings–Mack statistic p-value comparing median value for each parameter over days.

### Effect of Eurartesim^®^ on QTcF—statistical analysis

The final model included both random intercept and random slope (including random slope in a model gave the LR test of 5.66, p-value 0.03). The mean QTcF peaked on day 3 after the last dose of Eurartesim^®^ but decreased thereafter to the value close to baseline on day 7 (Fig 3). The mean QTcF on day 3 before treatment, day 3 after treatment and on day 7 were 12, 22 and 4 higher than that on Day 1 (p-value < 0.001) Table 5. The mean QTcF in children who are 5—< 12 years was higher by 13 ms (95% confidence interval 10.1, 15.0) than the mean QTcF in children whose age were 6 months to 5 years. Similarly, the mean QTcF in older children was about 20 higher than the mean QTcF in counterpart children who are 6 months to 5 years (Table 5). Adults (aged 18 and above) presented with the mean QTcF of 0.8 (95% confidence interval: -4.4, 2.9) lower than in children age between 12 to less than 18 years.

**Table 5:** Country-wise parameter estimates from linear mixed model.

Parameter	ALL		Ghana	Burkina Faso	Mozambique	Tanzania
	Estimate (SE)	95% CI	Estimate (SE)	Estimate (SE)	Estimate (SE)	Estimate (SE)
**Constant**	386.4 (1.6)**	383.3, 389.6	388.2 (2.3)**	388.8 (2.8)**	370.4 (13.2)**	377.0 (3.6)**
**6 months—< 5 years (reference)**						
**5—< 12 years**	12.6 (1.3)**	10.1, 15.0	13.8 (1.8)**	10.9 (2.1)**	22.2 (13.1)	11.4 (3.3)*
**12—< 18 years**	20.0 (1.8)**	16.5, 23.5	23.4 (2.6)**	19.7 (4.2)**	27.9 (13.7)*	18.6 (4.3)**
**18+ years**	19.2 (1.8)**	15.8, 22.7	23.3 (2.8)**	25.5 (4.1)**	32.2 (14.3)*	7.3 (3.7)‡
**Ghana (reference)**						
**Burkina Faso**	-2.5 (1.2)*	-5.0, -0.1	-	-	-	-
**Mozambique**	-3.8 (1.7)*	-7.1, -0.5	-	-	-	-
**Tanzania**	-10.8 (1.5)**	-13.7, -7.9	-	-	-	-
**Day 1 (reference)**						
**Day 3 pre-treatment**	12.0 (0.7)**	10.6, 13.4	14.2 (0.9)**	4.9 (1.1)**	24.7 (2.6)**	11.0 (2.0)**
**Day 3 after treatment**	22.1 (0.7)**	20.7, 23.5	27.5 (0.9)**	11.9 (1.1)**	44.2 (2.6)**	12.5 (2.0)**
**Day 7**	3.7 (0.7)**	2.3, 5.1	3.5 (0.9)**	1.5 (1.1)	11.2 (2.7)**	3.4 (2.1)
**Bilirubin total**	-0.2 (0.0)**	-0.2, -0.1	-0.2 (0.0)**	-0.2 (0.1)*	-0.1 (0.1)	-0.0 (0.1)
**Creatinine**	0.0 (0.0)	0, 0.1	-0.1 (0.0)	0.1 (0.1)	-0.2 (0.2)	0.1 (0.0)**

** P-value < 0.001,

* p-value < 0.05,

^‡^ borderline significant.

Country-wise the results of Mozambique site, children who were between 5 and 12 years had the mean QTcF of 22 ms more than children aged 6 months to 5 years. Similarly in Tanzania adults (18+ years) had the mean QTcF of 7 ms more than the youngest children at the end of their treatment course although this was on borderline significant. Patients in Burkina Faso, Mozambique and Tanzania had significant lower mean QTcF than patients in Ghana by an average of 3, 4 and 11 ms respectively (Table 5).

Children aged between 12 and 18 years had a mean QTcF of 7.5 (95% confidence interval: 4.4, 10.6) ms which was greater than children aged between 5 and 12 years. The mean QTcF in patients 18 years and above was 6.7 (95% confidence interval: 3.7, 9.6) ms higher than children who are 5 to less than 12 years (results not in tables).

On day 7 the mean QTc in male adults was slightly higher than that at baseline on day 1 by nearly 2 (95% confidence interval. -1.1, 4.5) ms [after having peaked with nearly 17 (13.6, 19.6) ms on day 3] as compared to counterpart adult females. In children, boys aged between 6 months and 5 years had significantly 5 (95% confidence interval 1.6, 8.6) higher mean QTcF compared to girls of the same age (Table 6).

**Table 6:** Age category based parameter estimates from linear mixed model.

Parameter	6 months—< 5 years		5—< 12 years		12—< 18 years		18+ years	
	Estimate (SE)	95% CI	Estimate (SE)	95% CI	Estimate (SE)	95% CI	Estimate (SE)	95% CI
**Constant**	380.4 (3.1)**	374.4; 386.4	396.2 (2.6)**	391.2; 401.2	406.3 (4)**	398.5; 414.1	409.8 (4.2)**	401.6; 417.9
**Ghana (reference)**								
**Burkina Faso**	-1.4 (2)	-5.4; 2.6	-4.2 (1.9)*	-7.9; -0.5	-3.9 (4)	-11.8; 3.9	4.8 (3.2)	-1.5; 11
**Mozambique**	-8.7 (11.1)	-30.4; 13.1	-2.7 (2.3)	-7.1; 1.8	-7.2 (3.4)*	-13.9; -0.5	-3.1 (4.1)	-11.1; 4.9
**Tanzania**	-9.9 (3.1)*	-16; -3.9	-8.6 (2.3)**	-13.2; -4.1	-8.8 (3.4)*	-15.4; -2.1	-15.8 (3.4)**	-22.4; -9.2
**Day 1 (reference)**								
**Day 3 pre-treatment**	11.1 (1.4)**	8.3; 13.9	13.9 (1.1)**	11.8; 16	14.1 (1.9)**	10.4; 17.9	7.3 (1.5)**	4.4; 10.3
**Day 3 after treatment**	18.6 (1.4)**	15.8; 21.4	24.5 (1.1)**	22.4; 26.6	28.6 (1.9)**	24.9; 32.4	16.6 (1.5)**	13.6; 19.6
**Day 7**	3.2 (1.5)*	0.3; 6	4.1 (1.1)**	1.9; 6.3	5.8 (2)*	2; 9.7	1.9 (1.5)	-1.1; 4.9
**Female (Reference)**								
**Male**	5.1 (1.8)*	1.6; 8.6	1.7 (1.4)	-1.2; 4.5	-0.9 (2.5)	-5.8; 4	-12.6 (2.8)**	-18.1; -7
**Bilirubin total**	-0.2 (0.1)*	-0.3; 0	-0.2 (0)**	-0.3; -0.1	-0.2 (0.1)*	-0.3; 0	0 (0.1)	-0.2; 0.2
**Creatinine**	0.2 (0.1)*	0; 0.3	0.1 (0)	0; 0.1	0 (0)	-0.1; 0.1	0 (0)	0; 0.1

** P-value < 0.001,

* p-value < 0.05.

Mean extreme QTcF prolongation from baseline was highest (60 ms) on day 3 after the last dose and lowest on the same day (33 ms) before last dose. On day 7 after treatment there was a downturn in the mean QTcF as compared to baseline which was recorded as 47 ms. In total we observed 79 (7%) extreme mean QTcF prolongation events from baseline which were also not clinically significant. The distribution of the extreme QTcF prolongations was 15 (19%), 58 (73%) and 6 (8%) recorded on day 3 before drug intake, day 3 post drug intake and on day 7 after treatment respectively. Almost a half of all extreme QTcF prolongation events that occurred were grade 3, (37/79) whereas male accounted the majority (33/37) and children aged 5-<12 years were more than a half of all grade 3 extreme prolongation cases (n = 19). Male patients had majority of QTcF prolongation events than female throughout the treatment period (Table 7).

**Table 7:** QTcF extreme prolongation from baseline to Day 3 and Day 7.

Attribute	Mean (SD) increase from baseline	Events n (%)	Grade 1 n (%)	Grade 2 n (%)	Grade 3 n (%)
**Day 3 before dose**					
**Total**	33.4 (18.9)	15 (1.3)	7 (0.6)	5 (0.4)	3 (0.3)
**Male**	31.4 (18.9)	13 (2.4)	7 (1.3)	4 (0.7)	2 (0.4)
**Female**	46.2 (17.7)	2 (0.3)	0	1 (0.2)	1 (0.2)
**6 months—< 5 years**	-	0	0	0	0
**5—< 12 years**	34.8 (18.0)	6 (1.2)	2 (0.4)	3 (0.6)	1 (0.2)
**12—< 18 years**	41.3 (19.2)	6 (4.2)	2 (1.4)	2 (1.4)	2 (1.4)
**18 + years**	14.8 (7.5)	3 (1.4)	3 (1.4)	0	0
**Day 3 after dose**					
**Total**	59.9 (31.1)	58 (5.1)	7 (0.6)	14 (1.2)	37 (3.2)
**Male**	61.6 (32.3)	50 (9.1)	5 (0.9)	12 (2.2)	33 (6.0)
**Female**	49.3 (21.4)	8 (1.3)	2 (0.3)	2 (0.3)	4 (0.7)
**6 months—< 5 years**	66.0 (13.8)	6 (2.1)	0	1 (0.4)	5 (1.7)
**5—< 12 years**	64.0 (35.7)	30 (5.9)	3 (0.6)	8 (1.6)	19 (3.8)
**12—< 18 years**	56.1 (28.2)	15 (10.5)	1 (0.7)	4 (2.8)	10 (7.0)
**18 + years**	45.6 (24.9)	7 (3.3)	3 (1.4)	1 (0.5)	3 (1.4)
**Day 7** **after treatments**					
**Total**	46.9 (27.8)	6 (0.5)	1 (0.1)	3 (0.3)	2 (0.2)
**Male**	40.0 (25.4)	5 (0.9)	1 (0.2)	3 (0.5)	1 (0.2)
**Female**	79.7 (0)*	1 (0.5)	0	0	1 (0.2)
**6 months—< 5 years**	43.3 (0)*	1 (0.4)	0	1 (0.4)	0
**5—< 12 years**	51.6 (20.9)	3 (0.5)	0	2 (0.4)	1 (0.2)
**12—< 18 years**	-	0	0	0	0
**18 + years**	41.8 (27.8)	2 (1.0)	1 (0.5)	0	1 (0.5)

* Only one subject had prolonged QTcF (therefore SD not calculated).

## Discussion

Whereas use of ECG for cardiotoxicity evaluation of other ACTs has been well detailed in literature, this study gives additional evidence of safety of DHA/PPQ (Eurartesim^®^) evaluated by the incorporation of ECG and laboratory measurements of changes in patients treated with Eurartesim^®^. DHA/PPQ is one of the antimalarials that has shown a good efficacy against *P*.*falciparum* infection [2,21,22,23,24,25]. Acute *P*.*falciparum* malaria fever, especially the severe form is also associated with tachyarrhythmia a manifestation form of shortening of QT interval [26] of which the recovery is characterized by QT prolongation [27]. In this study the mean QTcF at baseline was within normal range, almost in all age categories of patients that participated who received Eurartesim^®^. There were prolongations in the mean QTcF during the course of treatment with Eurartesim^®^ that was provided on Day 1 through Day 3. These changes were recorded in 79 cases of extreme QTcF prolongation that reverted to initial baseline QTc values in most patients on Day 7. In this study, QTc maximum prolongation was observed on Day 3 corresponding to what would be considered onset of peak plasma concentration of piperaquine following the last dose administration [11]. Peaks of QTcF were recorded alongside minimum laboratory biochemistry values (except for potassium which was in reverse) that were also measured low during treatment as compared to the maximum values that were recorded at baseline with some restorations on day 7. There was sound difference in the mean values of QTcF prolongation between baseline and during treatment among 79 subjects. Although the mean QTcF was such extreme these changes were not associated with significant clinical adverse condition. The occurrences of extreme QTc prolongation we have observed were mostly in children who are 5 to < 12 years devoid of any sound clinical cardiogenic adverse condition might indicate the possibility of a wider therapeutic threshold in this group of children relative to younger children or adults was warranted. Another line of deduction would be that probably this prolongation in the mean QTcF intervals and the recovery thereof reflects the recovery from acute malaria episode [27]. Our study was not powered to look at this detailed difference. Moreover, in this study we did not see any complete restoration of QTcF on day 7 the phenomena that would have reflected the residual piperaquine in the blood of which required an extended time of observation to ascertain the full restoration. Despite the modest prolongation that was observed in this study as compared to baseline values, this prolongation is of little clinical relevance as it did not exacerbate any clinical sound cardiac manifestation of arrhythmia [5]. In the literature extreme QTc prolongation following drug administration are often associated with fatal form of arrhythmia known as *torsades de pointes*. In our study QTcF prolongation that we observed did not worsen to *torsades de pointes* most probably due to the therapeutic dose that we used being within safety margin of therapeutic thresholds. Reversible ECG changes from baseline on day 1 as demonstrated through the recession of mean QTc interval values recorded on day 7 suggests the safety of the optimal therapeutic dose of Eurartesim^®^ among African patients with uncomplicated malaria is warranted. One would suggest that perhaps we did not see untoward extreme maximum QTc prolongation in the study population because all patient with the history of cardiac problems were excluded at baseline. This position has some bearing and cannot go without comment. Our approach was to exercise utmost safety precautions and this might have enabled us to avoid any unnecessary torsadogenic events that might resulted, had we included-pro-arrhythmic patients. These findings also reflect the classic overall presentation of plasma concentration levels that are expected to decline around day 7 corresponding QTc recovery [11]. In the by-country comparison however, one of the possible explanations for the modest QTc prolongation that was noted in some patients from Ghana as compared to the rest might also be due to other factors that were not investigated in this study such as genetic or dietary factors.

In classic QT interval evaluation studies, positive controls are often used to test the study’s ability to detect the endpoint of interest, i.e., the QTc interval above the maximum normal thresholds of ≥480ms. In the current study our design was destined to detect rare SAE due to cardiotoxicity from a therapeutic dose of Eurartesim^®^ in a large population exposure. The use of positive control in a post licensure surveillance study was therefore not envisaged. The design employed by this study aimed to measure the QTc effect of Eurartesim^®^ currently used after subsequent dosing through a well-established regimen of days 1–3 with subsequent ECG changes as compared to the ones recorded at baseline. It is for this reason that modeling of ECG outcome was crucial to quantify the finding of no QT effect if the size relative to the control is evident had the control been considered. It is thus imperative to note here that any value of changes in QTc that have been modelled in this study represented a crucial addition piece of evidence on changes in QTc during treatment of uncomplicated malaria with Eurartesim^®^. Blood chemistry and haematology exhibited temporal changes that remained within clinical thresholds not requiring any interventions. This phenomena also confirms that limited therapeutic Eurartesim^®^ toxicity was equally tolerated across all parameters that we carried out the measurements. It can therefore modestly be asserted that Eurartesim^®^ in current therapeutic dose has limited clinical toxicity consistent with QTc intervals prolongation when the drug is used in none-pro-arrhythmic uncomplicated malaria patients.

### Limitations

One of the limitations of our results is the lack of direct extrapolation to people who are prone to cardiac problems due to our set exclusion criteria. We restricted enrolment of all subjects that had history of predisposing cardiac conditions for arrhythmia and those contraindicated to products that are known to prolong the QTc interval [9]. Our choice to exclude subjects of this type is in line with most post-marketing safety evaluation settings that limit uncontrolled exposure to subjects with visible risk under the evaluation.

The second limitation is the fact that QT prolongation reported by our study was a modest increase that could have been due to recovery from acute malaria. Again this setback would have been dealt by comparison with other antimalarial treatment with established lack of cardiotoxicity. Our study was designed to assess safety of antimalarial in a post licensure environment and the findings we have reported correspond to this setting. Lastly is the limitation that the modest difference in QTc changes we have observed among cases, cannot be compared with subjects that would have been included but in this case excluded at baseline for reasons of predisposing cardiac conditions for arrhythmia or who took other antimalarial that cause QT prolongation. This was based on history, as we did not investigate at baseline the QTc status in all individuals that were screened for inclusion. Screening of this type is in particular not feasible to be implemented in the routine health care setting. We also confirm that our findings may not be extrapolated to patients who are predisposing to cardiac conditions for arrhythmia or those who might have taken antimalarial that cause QT prolongation because they were excluded.

This paper reports on a study that recorded few other adverse events that were not directly related to ECG prolongation as was reported previously by the co-author of this paper elsewhere [28]. It was recorded that a modest non-clinically significant increase in QT interval which also normalized after treatment without involving any intervention was seen. These findings corroborates the widely documented *invitro* animal studies in which the possible lack of huge prolongation or torsadogenic cardiac risk in human when DHA/PPQ is used could be related with the current dose as being optimal therapeutic dose [1]. The findings fill the existing gap in evidence to warrant a wider use of few available highly effective ACT in malaria endemic settings.

## Conclusion

This study has demonstrated the methodology of field based ECG recording being deployed in remote health facilities for drug safety evaluation. The study has also demonstrated that despite long half-life of piperaquine no clinically significant cardiovascular or metabolic effects were observed among African patients with uncomplicated *P*.*falciparum* malaria. Therefore we found no evidence that dihydroartemisinin/piperaquine administered in therapeutic doses in patients with uncomplicated malaria and no predisposing cardiac conditions in Africa was associated with adversely clinical significant QTc prolongation.

## Supporting Information

S1 Checklist (PDF)

S1 Protocol (PDF)
